# A Critical Overview of Systematic Reviews and Meta-Analyses on Acupuncture for Poststroke Insomnia

**DOI:** 10.1155/2020/2032575

**Published:** 2020-10-09

**Authors:** Jinke Huang, Manli Wu, Simin Liang, Xiaohui Qin, Min Shen, Jiansen Li, Yong Huang

**Affiliations:** ^1^The Second Clinical Medical College of Guangzhou University of Chinese Medicine, Guangzhou, Guangdong Province 510120, China; ^2^Department of Neurology, Guangdong Provincial Hospital of Chinese Medicine, Guangzhou, Guangdong Province 510120, China; ^3^School of Traditional Chinese Medicine, Southern Medical University, Guangzhou, Guangdong Province 510515, China

## Abstract

**Objectives:**

Acupuncture has increasingly been used for insomnia relief after stroke. We aimed to evaluate the methodological quality and summarize the evidence regarding the effectiveness of acupuncture for poststroke insomnia (PSI) from systematic reviews/meta-analyses (SRs/MAs).

**Methods:**

Eight databases were searched from inception through August 23, 2020. SRs/MAs on acupuncture treatment for PSI were included. Methodological quality assessment was performed using Assessing the Methodological Quality of Systematic Reviews 2 (AMSTAR-2), and evidence quality assessment was performed using the Grading of Recommendations, Assessment, Development, and Evaluation (GRADE).

**Results:**

Six SRs/MAs on acupuncture treatment for PSI were included. The AMSTAR-2 showed that the methodological quality of all included SRs/MAs was rated as critically low. According to the evaluation results of GRADE, 38.9% (7/18) of outcomes were rated as very low-quality evidence, 22.2% (4/18) were low-quality evidence, and 8.9% (7/18) were moderate-quality evidence. Descriptive analysis results showed that acupuncture was an effective treatment modality for PSI.

**Conclusions:**

All included reviews indicated that acupuncture was more effective than the control group for the treatment of PSI, but the credibility of the results is limited owing to the generally low methodological and evidence quality of the included SRs/MAs. More high-quality evidence is needed to determine whether acupuncture is more effective than other treatments.

## 1. Introduction

Worldwide, stroke is the second leading cause of death and the third-largest source of disability [1]. Insomnia is one of the most common poststroke complications, affecting approximately 40% to 60% of stroke patients [2]. Although a large number of chronic poststroke insomnia (PSI) patients are produced, insomnia continues to be the most unrecognized modifiable risk factor for stroke [3]. Untreated PSI causes cognitive dysfunction, altered mood, and fatigue and may impede stroke rehabilitation, lengthen hospital stay, influence stroke outcomes, and increase the possibility of stroke recurrence [4]. The primary pharmacological treatments for PSI are benzodiazepines, nonbenzodiazepine sedatives, and melatonin agonists. Although effective pharmacological treatments are available, their significant side effects have limited their clinical applications and long-term use [5]. The mainstay of nonpharmacological interventions for insomnia is cognitive behavioral therapy. However, cognitive behavioral therapy often results in an acute reduction in sleep time during the first few weeks of treatment, which means that sleep quality improvement with cognitive behavioral therapy requires long-term implementation [6]. Therefore, faced with the limitations of currently available PSI treatments, many people are interested in complementary and alternative medicine.

Among the complementary treatment modalities, acupuncture is one of the safe and most popular treatments [7]. According to the theory of traditional Chinese medicine (TCM), acupuncture provides overall coordination to help the body achieve a state of relative equilibrium of yin and yang, restoring its physiological function and regulating the sleep-wake cycle [8]. Systematic reviews (SRs)/meta-analyses (MAs) are considered the gold standard for assessing the effects of health care interventions, but their methodology must comply strictly with a series of guidelines, so as to minimize the possibility of bias in answering a specific research question [9]. Furthermore, the generation of assessment tools such as the Assessing the Methodological Quality of Systematic Reviews 2 (AMSTAR-2) [10] and the Grades of Recommendation, Assessment, Development, and Evaluation (GRADE) [11] have also played prominent roles in designing, reporting, and assessing high-quality SRs/MAs. However, not all authors of SRs/MAs have maintained strict adherence to the criteria, which has led to low-quality reviews and difficulty in providing convincing conclusions [12]. A literature search yielded several published systematic reviews (SRs)/meta-analyses (MAs) that have reported that acupuncture is efficacious for PSI, but their methodological and evidence quality have not been evaluated. Therefore, we composed an overview to summarize the evidence on the effectiveness of acupuncture for PSI.

## 2. Materials and Methods

### 2.1. Inclusion and Exclusion Criteria

The inclusion criteria were as follows: (a) study design: SRs/MAs based on randomized controlled trials (RCTs); (b) participants: the participants had PSI diagnosed according to any authoritative diagnostic criteria, no restrictions on sex, age, race, onset time, or the source of cases; (c) intervention: acupuncture therapy (manual acupuncture, electroacupuncture, or auricular acupuncture, etc.) versus conventional medication (CM) or acupuncture therapy combined with CM versus CM alone; and (d) outcomes: effective rate, sleep parameters, sleep efficiency, scales, or index for sleep quality evaluation, adverse effects.

The exclusion criteria were as follows: (a) animal studies; (b) network MAs, overviews, and narrative reviews; and (c) studies in which the required data were unavailable.

### 2.2. Search Strategy

Literature searches were conducted in PubMed, EMBASE, the Cochrane Library, Web of Science, China National Knowledge Infrastructure (CNKI), Wanfang Database, Chongqing VIP, and SinoMed from their inceptions to March 28, 2020, and we conducted an updated search on August 23, 2020, to provide more up-to-date and comprehensive evidence. Details of the search strategy for each database are presented in Additional file 1. There were no restrictions on language, publication status (including gray literature), or publication country.

### 2.3. Eligibility Assessment and Data Extraction

Two reviewers (SM-L and M-S) independently screened the titles and abstracts of the retrieved citations, evaluated the potential full texts, and determined the eligibility of the reviews. Disagreements were resolved by consensus or arbitration by a third reviewer (Y-H).

Two independent reviewers (SM-L and M-S) extracted the following data: the first author, publication year, country, numbers of included trials and participants, quality assessment methods, interventions, outcomes, and the main results of each included review.

### 2.4. Methodological Quality Assessment

The Assessing the Methodological Quality of Systematic Reviews 2 (AMSTAR-2) tool [10] was used by two independent reviewers (XH-Q and M-S) to assess the methodological quality of the included SRs/MAs. The AMSTAR-2 tool contains 16 items, and seven of them are critical items. Details of the 16 items are presented in Additional file 2. The quality of each item (especially the critical items) should be fully considered and categorized into four levels, namely, high, moderate, low, and critically low. Disagreements were resolved by consensus or arbitration by a third reviewer (Y-H).

### 2.5. GRADE Scoring

The Grades of Recommendation, Assessment, Development, and Evaluation (GRADE) [11] system based on the GRADEpro software (http://ims.cochrane.org/revman/gradepro) was used to assess the evidence quality of the outcome measures of the included SRs/MAs. The following criteria were taken into account: the risk of bias, inconsistencies, indirectness, inaccuracy, and publication bias. The evidence quality was evaluated by two reviewers (XH-Q and M-S) independently, and disagreements were resolved by consensus or arbitration by a third reviewer (Y-H).

## 3. Results

### 3.1. Literature Selection

**Figure 1:**
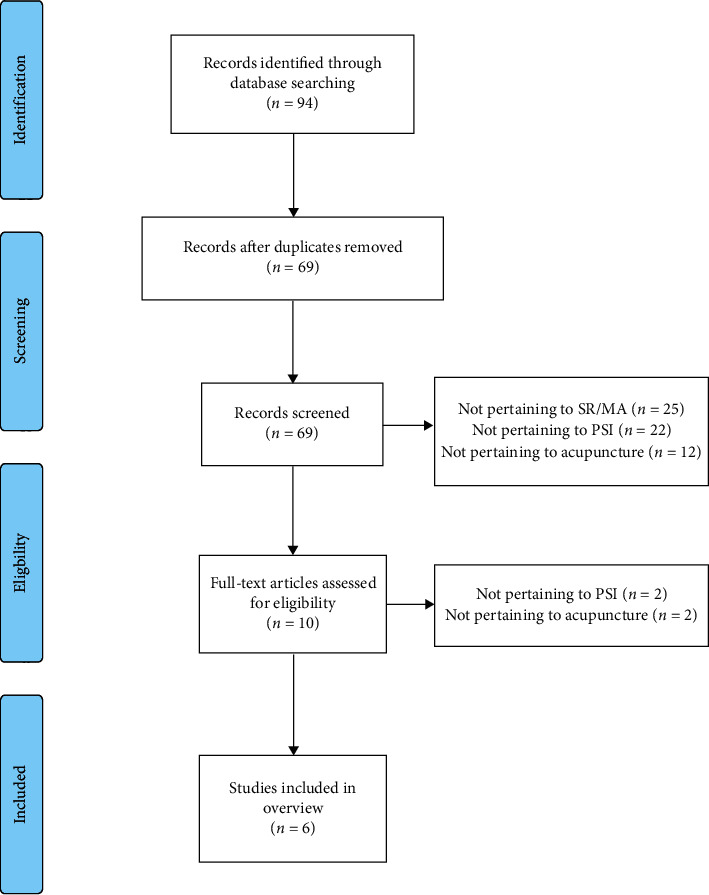
Flowchart of the literature search.

The literature search retrieved 94 potentially relevant studies, and after removing the duplicates and performing titles/abstracts screening, we excluded 84 studies. The remaining ten studies were retrieved for full-text assessment. We excluded four studies because two of these did not focus on acupuncture and two did not focus on PSI. Finally, six studies [13–18] were included in this overview. Details of the literature selection can be found in Figure 1.

### 3.2. Study Characteristics

The descriptive characteristics of the included SRs/MAs are presented in Table 1. The SRs/MAs were published between 2015 and 2020 and included between seven and 35 trials each with sample sizes ranging from 435 to 2866 subjects. All SRs/MAs included only RCTs. These SRs/MAs were mainly published by authors from East Asia (five [14–18] from China and one [13] from Korea); five [14–18] were published in Chinese and one [13] in English; four [13–15, 17] were published in journals, and two [16, 18] were master's degree papers in China. The intervention measures were mostly acupuncture therapy or acupuncture plus other therapies (e.g., auricular acupressure) in the treatment group and CM, and sham acupuncture in the control group. Five SRs/MAs [13–17] used the Cochrane risk of bias criteria to evaluate the quality of the trials, and the remaining one [18] used the Jadad scale.

### 3.3. Methodological Evaluation

After evaluation of the methodological quality by the AMSTAR-2 tool, all included SRs/MAs were rated to be of critically low quality. The critical items that were most frequently lacking were item 2 (lack of the protocol being registered before review commencement (6/6, 100%)), item 4 (lack of a search strategy for each database (2/6, 33.3%)), item 7 (lack of a list of excluded trails (2/6, 33.3%)), and item 15 (lack of assessment of the present and likely impact of publication bias (1/6, 16.7%)). The breakdown of the AMSTAR-2 assessment for each review is presented in Table 2.

### 3.4. Evidence Quality Evaluation

After evaluation of the evidence quality of the 18 outcome measures by the GRADE system, 38.9% (7/18), 22.2% (4/18), and 38.9% (7/18) were rated to be of moderate, low, and critically low quality, respectively. The key factors affecting the evidence quality included the study limitations within the original trials, inconsistency, imprecision, and the possibility of publication bias. The details of the GRADE evaluation are shown in Table 3.

### 3.5. Outcomes and Efficacy Evaluation

#### 3.5.1. Effective Rate

Five reviews [14–18] analyzed the effective rate of acupuncture for PSI. In four reviews [14–16, 18], acupuncture appeared to be more effective than CM for the treatment of PSI. Additionally, according to the results of other reviews [14, 17, 18], acupuncture plus other therapy was more effective than CM alone. In one review that used the cure rate to compare the effects of acupuncture with those of CM, acupuncture was observed to have a significant difference in reducing PSI.

#### 3.5.2. PSQI Score

Two reviews [13, 15] used the Pittsburgh Sleep Quality Index (PSQI) score to compare the effects of acupuncture with those of CM. It was found that acupuncture was more effective than CM for the treatment of PSI. In one review [17] that used the PSQI score to compare the effects of acupuncture plus auricular acupressure with those of CM, the results indicated that the combined treatment was superior to CM alone in terms of improvement in daytime dysfunction, but there was no statistical difference between different treatments in terms of improvement in sleep onset latency and sleep time.

#### 3.5.3. ISI and AIS Score

One review [13] comparing the effects of intradermal acupuncture with those of sham acupuncture used the Athens Insomnia Scale (ISI)and the Insomnia Severity Index (AIS). In this review, acupuncture had a significant effect on PSI relative to sham, as assessed by both by the ISI and the AIS.

#### 3.5.4. TCM Syndrome Score

Two reviews [13, 15] comparing the effects of acupuncture with those of CM used the TCM syndrome score. Of these reviews, the results of Lee and Lim [13] showed that acupuncture had a better effect than CM, while the other review [15] reported there was no statistical difference between the different treatments.

#### 3.5.5. Depression and Anxiety

One review [13] comparing the effects of acupuncture with those of CM used the Self-Rating Anxiety Scale (SAS) or the Self-Rating Depression Scale (SDS). According to the results, no significant differences in scores for depression and anxiety were found between the different treatments.

## 4. Discussion

Insomnia is a common complication following stroke, often interfering with activity, recovery, and rehabilitation. As a widely used therapy in China for poststroke rehabilitation, the effect of acupuncture in improving sleep quality in stroke patients has attracted the attention of researchers. Currently, there have been multiple SRs/MAs of relevant clinical trials to explore the effect of acupuncture for PSI. Based on a comprehensive research method, we conducted a comprehensive assessment of SRs/MAs with varying quality to further explore the reliability of the results, in order to provide a basis for future related research by clinical and scientific researchers.

### 4.1. Summary of Quality

The results of the AMSTAR-2 evaluation show that each SR/MA had different deficiencies in terms of its design, registration, data extraction, and analysis. None of the SRs/MAs had registered a preliminary design protocol, which may have resulted in a larger adjustment of the study process than expected, increasing the risk of bias and affecting the rigor of the SR/MA. For an SR/MA, a protocol should be designed and registered in advance to ensure that the study implementation process is methodical. Although all of the reviews had conducted literature searches in more than one database, two [14, 17] only provided search keywords but no specific search strategy, which may result in publication bias and affect the conclusion's reliability. A full and comprehensive literature search is quite critical for secondary literature studies like SRs/MAs. None of the SRs/MAs provided a complete list of potential studies with reasons for the exclusion of each, which may affect the reliability of the results. Although the majority of the published SRs/MAs were journal articles with word count and format restrictions, providing a list of excluded articles could more strongly prove the rigor of the literature screening process. Two [16, 18] SRs/MAs did not report funding resources or state their conflicts of interest, which may increase study reporting bias because the results of business-funded studies might be biased towards the funder. One review [17] did not consider publication bias and provided explanations for its possible sources when the authors interpreted or discussed the study results, which may affect the authenticity of the final results. The deficiencies mentioned above are the main reasons for the critically low methodological quality rating of the included SRs/MAs.

The results of the GRADE evaluation indicated that the current quality of evidence was not satisfying. Of these 18 outcome measures, seven, four, and seven were rated to be of moderate, low, and critically low quality, respectively. The key factors affecting the evidence quality included limitations, inconsistency, imprecision, and the possibility of publication bias. The study limitations within the original trials were the main factors in the low quality of all of the evidence. Most of the RCTs included in the SRs/MAs had an unclear risk of bias for blinding, random sequence generation, and allocation concealment. Although blinding of the therapists who perform acupuncture would be difficult, blinding of patients, other care providers, and outcome assessors should be attempted in order to minimize the performance and assessment bias. Reduced evidence quality due to inconsistencies may originate from substantial clinical and methodological variations in the included RCTs, which can be avoided by standardizing the inclusion and exclusion criteria and standardizing the literature screening process. Additionally, imprecision and publication bias also lead to downgrading of the quality of evidence, which may be related to implausible study designs. These deficiencies in the evidence quality indicate that the conclusions of the SRs/MAs may differ from the true results and thus cannot provide a scientific basis for clinicians.

Assessment of various aspects of the quality of the included SRs/MAs identified common areas for improvement, and thus, the practice of using the widely accepted tools like AMSTAR-2 and GRADE for designing, reporting, and assessing SRs/MAs needs to be encouraged and advocated, thereby providing more convincing evidence based on their findings. The core reason for the low quality of the SRs/MAs may be that a significant proportion of acupuncture SRs/MAs researchers may not receive sufficient evidence-based medicine education [19]. Therefore, we should strengthen and improve evidence-based medicine education in university and medical school and continuing education through TCM, especially regarding the use of the Cochrane Handbook and some widely accepted tools like AMSTAR-2 and GRADE. Furthermore, AMSTAR-2 and GRADE were introduced in 2004 and 2017, respectively. It is clear that they both existed prior to the publication of the included SRs/MAs. This phenomenon suggests a lack of normative awareness among the authors of SRs/MAs, as most weaknesses can be avoided or reduced if authors of SR/MAs could increase their awareness of standard methodology and learn how to use the AMSTAR-2 and GRADE tools before the commencement of SRs/MAs.

### 4.2. Limitations

This study is the first overview of SRs/MAs using AMSTAR-2 and GRADE to explore the evidence about using acupuncture for PSI. Based on the current results, the methodological and evidence quality of the included SRs/MAs were not satisfying, so our study cannot draw firm conclusions about the use of acupuncture for PSI. Additional high-quality randomized trials and MAs are necessary to determine whether acupuncture is more effective than other treatments.

## 5. Conclusion

All included SRs/MAs suggested that acupuncture was more effective than CM or sham acupuncture in the treatment of PSI; however, this conclusion was limited due to the generally low methodological and evidence quality of the included studies. The practice of using widely accepted tools like AMSTAR-2 and GRADE for designing, reporting, and assessing SRs/MAs needs to be encouraged and advocated, thereby providing more convincing evidence based on their findings.

## Figures and Tables

**Table 1:** Characteristics of the included reviews.

Studies	Country	Trials (sample size)	Treatment intervention	Control intervention	Quality assessment tool	Overall conclusion
Lee and Lim 2016 [13]	Korea	13 (1051)	AT	CM; sham AT	Cochrane criteria	Acupuncture could be effective for treating PSI. However, further studies are needed to confirm the role of acupuncture in the treatment of this disorder.

Nie et al. 2020 [14]	China	19 (1535)	AT; AT + other therapies	CM	Cochrane criteria	Acupuncture treatment for PSI was effective.

Zhang et al. 2019 [15]	China	34 (2698)	AT	CM	Cochrane criteria	The clinical efficacy of acupuncture for PSI was better than that of western medicine.

Wu 2019 [16]	China	35 (2866)	AT	CM	Cochrane criteria	Acupuncture was effective in the treatment of PSI and had a certain clinical value.

Fu et al. 2018 [17]	China	7 (435)	AT + AA	CM	Cochrane criteria	The effect of acupuncture combined with auricular acupressure in the treatment of PSI was better than that of the control group with no adverse reactions.

Liang [18] 2015	China	20 (1547)	AT; AT + other therapies	CM	Jadad score	The clinical effect of treating PSI with acupuncture was better than that of medicine.

AT: acupuncture therapy; CM: conventional medication; AA: auricular acupressure.

**Table 2:** Results of the AMSTAR-2 assessments.

Studies	AMSTAR-2																Overall quality
	Q1	Q2	Q3	Q4	Q5	Q6	Q7	Q8	Q9	Q10	Q11	Q12	Q13	Q14	Q15	Q16	
Lee and Lim 2016 [13]	Y	PY	Y	Y	Y	Y	N	Y	Y	Y	Y	Y	Y	Y	Y	Y	CL
Nie et al. 2020 [14]	Y	PY	Y	PY	Y	Y	N	Y	Y	Y	Y	Y	Y	Y	Y	Y	CL
Zhang et al. 2019 [15]	Y	PY	Y	Y	Y	Y	N	Y	Y	Y	Y	Y	Y	Y	Y	Y	CL
Wu 2019 [16]	Y	PY	Y	Y	Y	Y	N	Y	Y	N	Y	Y	Y	Y	Y	N	CL
Fu et al. 2018 [17]	Y	PY	Y	PY	Y	Y	N	Y	Y	Y	Y	Y	Y	Y	N	Y	CL
Liang [18] 2015	Y	PY	Y	Y	Y	Y	N	Y	Y	N	Y	Y	Y	Y	Y	N	CL

Y: yes; PY: partial yes; N: no; CL: critically low; L: low; H: high.

**Table 3:** Certainty of evidence quality evaluation.

Studies	Interventions	Outcomes	Studies (participants)	Limitations	Inconsistency	Indirectness	Imprecision	Publication bias	Quality
Lee and Lim 2016 [13]	AT versus CM	PSQI score	6 (385)	−1^①^	−1^②^	0	0	0	Low
		TCM syndrome score	7 (497)	−1^①^	−1^②^	0	0	0	Low
	AT versus sham AT	ISI score	2 (82)	−1^①^	0	0	−1^③^	−1^④^	Very low
		AIS score	2 (82)	−1^①^	0	0	−1^③^	−1^④^	Very low

Nie et al. 2020 [14]	AT versus CM	Effective rate	12 (786)	−1^①^	0	0	0	0	Moderate
	AT + other therapies versus CM	Effective rate	7 (749)	−1^①^	0	0	0	0	Moderate

Zhang et al. 2019 [15]	AT versus CM	Effective rate	26 (1993)	−1^①^	−1^②^	0	0	0	Low
		Cure rate	32 (2330)	−1^①^	0	0	0	0	Moderate
		PSQI score	27 (1764)	−1^①^	−1^②^	0	0	0	Low
		TCM syndrome score	2 (130)	−1^①^	−1^②^	0	−1^③^	−1^④^	Very low
		Scores for depression and anxiety	3 (190)	−1^①^	−1^②^	0	−1^③^	−1^④^	Very low

Wu 2019 [16]	AT versus CM	Effective rate	12 (803)	−1^①^	0	0	0	0	Moderate

Fu et al. 2018 [17]	AT + AA versus CM	Effective rate	7 (436)	−1^①^	0	0	0	0	Moderate
		Sleep onset latency	3 (180)	−1^①^	0	0	−1^③^	−1^④^	Very low
		Sleep time	3 (180)	−1^①^	0	0	−1^③^	−1^④^	Very low
		Daytime dysfunction	3 (180)	−1^①^	0	0	−1^③^	−1^④^	Very low

Liang 2015 [18]	AT versus CM	Effective rate	12 (1015)	−1^①^	0	0	0	0	Moderate
	AT + other therapies versus CM	Effective rate	5 (342)	−1^①^	0	0	0	0	Moderate

AT: acupuncture therapy; CM: conventional medicine; AA: auricular acupressure; PSQI: Pittsburgh Sleep Quality Index; TCM: traditional Chinese medicine; AIS: Athens Insomnia Scale; ISI: Insomnia Severity Index. ^①^The design of the experiment has a large bias in randomization, distributive concealment, or blinding; ^②^the confidence interval overlaps less, the heterogeneity test *P* is very small, and the *I*^2^ is larger; ^③^the confidence interval is not narrow enough; ^④^funnel graph asymmetry; fewer studies are included and there may be a greater risk of publication bias.

## Data Availability

All data generated or analyzed during this study are included within the article.

## Abbreviations

PSI: Poststroke insomnia
SR: Systematic review
MA: Meta-analysis
AMSTAR-2: Assessing the Methodological Quality of Systematic Reviews 2
GRADE: Grading of Recommendations, Assessment, Development, and Evaluation
RCTs: Randomized clinical trials
CNKI: China National Knowledge Infrastructure
CM: Conventional medication
TCM: Traditional Chinese medicine
PSQI: Pittsburgh Sleep Quality Index
ASI: Athens Insomnia Scale
ISI: Insomnia Severity Index
SAS: Self-Rating Anxiety Scale
SDS: Self-Rating Depression Scale.
